# 360 Health Analysis (H360)—A Comparison of Key Performance Indicators in Breast Cancer Management across Health Institution Settings in Portugal

**DOI:** 10.3390/curroncol30070451

**Published:** 2023-06-23

**Authors:** Inês Brandão Rego, Sara Coelho, Patrícia Miguel Semedo, Joana Cavaco-Silva, Laetitia Teixeira, Susana Sousa, Joana Reis, Rui Dinis, Fernando Schmitt, Noémia Afonso, José Luís Fougo, Francisco Pavão, Ricardo Baptista Leite, Luís Costa

**Affiliations:** 1 Institute of Health Sciences, Universidade Católica Portuguesa, Palma de Cima, 1649-023 Lisboa, Portugal; rego_ines@hotmail.com (I.B.R.); saramarinacoelho@gmail.com (S.C.); patricia.semedo@chln.min-saude.pt (P.M.S.); laetitiateixeir@gmail.com (L.T.); spsousa@icbas.up.pt (S.S.); pavaojf@gmail.com (F.P.); ricardo.baptistaleite@gmail.com (R.B.L.); luiscosta.oncology@gmail.com (L.C.); 2 Hospital de São João, Centro Hospitalar Universitário de São João, 4200-319 Porto, Portugal; joana11silvareis@gmail.com; 3 Instituto Português de Oncologia do Porto Francisco Gentil EPE, 4200-072 Porto, Portugal; 4 Hospital de Santa Maria, Centro Hospitalar Universitário Lisboa Norte, 1649-028 Lisboa, Portugal; 5 Instituto de Medicina Molecular-João Lobo Antunes, Faculdade de Medicina da Universidade de Lisboa, 1649-028 Lisboa, Portugal; 6 ScienceCircle-Scientific and Biomedical Consulting, 1600-369 Lisboa, Portugal; 7 Instituto de Ciências Biomédicas Abel Salazar, Universidade do Porto, 4050-313 Porto, Portugal; 8 Hospital do Espírito Santo de Évora, 7000-811 Évora, Portugal; ruidini@gmail.com; 9 Faculdade de Medicina da Universidade do Porto, 4200-319 Porto, Portugal; fschmitt@ipatimup.pt (F.S.); joseluis.fougo@chsj.min-saude.pt (J.L.F.); 10 Centro Hospitalar de Vila Nova de Gaia e Espinho, 4400-129 Vila Nova de Gaia, Portugal; noemia.afonso@netcabo.pt; 11 Centro de Mama, Centro Hospitalar Universitário de São João, 4200-319 Porto, Portugal; 12 Faculty of Health, Medicine and Life Sciences, Maastricht University, 6211 LK Maastricht, The Netherlands

**Keywords:** breast cancer, compliance, EUSOMA, health system, key performance indicator, management

## Abstract

Background: The increased focus on quality indicators (QIs) and the use of clinical registries in real-world cancer studies have increased compliance with therapeutic standards and patient survival. The European Society of Breast Cancer Specialists (EUSOMA) established QIs to assess compliance with current standards in breast cancer care. Methods: This retrospective study is part of H360 Health Analysis and aims to describe compliance with EUSOMA QIs in breast cancer management in different hospital settings (public vs. private; general hospitals vs. oncology centers). A set of key performance indicators (KPIs) was selected based on EUSOMA and previously identified QIs. Secondary data were retrieved from patients’ clinical records. Compliance with target KPIs in different disease stages was compared with minimum and target EUSOMA standards. Results: A total of 259 patient records were assessed. In stages I, II, and III, 18 KPIs met target EUSOMA standards, 5 met minimum standards, and 8 failed to meet minimum standards. Compliance with KPIs varied according to the type of hospital (particularly regarding diagnosis) and disease stage. Although small differences were found in KPI compliance among institutions, several statistical differences were found among treatment KPIs according to disease stage, particularly in stage III. Conclusions: This study represents the first assessment of the quality of breast cancer care in different hospital settings in Portugal and shows that, although most QIs meet EUSOMA standards, there is room for improvement. Differences have been found across institutions, particularly between oncology centers and general hospitals, in diagnosis and compliance with KPIs among disease stages. Stage III showed the greatest variability in compliance with treatment KPIs, probably related to the lower specificity of the guidelines in this disease stage.

## 1. Introduction

In 2020, female breast cancer had a global incidence of 2.3 million new cases (11.7% of all cancer cases), ranking as the most commonly diagnosed cancer [1]. The disease represents a substantial health burden for both the individuals and society.

Setting performance measures in cancer, known as key performance indicators (KPIs), is a highly recognized mechanism of monitoring and measuring the quality of care delivered in health centers and enables accurately comparing cancer centers and identifying areas for improvement. KPIs are increasingly becoming a requirement in the delivery of health care in most institutions globally [2].

The European Society of Breast Cancer Specialists (EUSOMA) provides a voluntary certification process for breast centers that ensures multidisciplinary care and minimum standards of care. EUSOMA defined quality indicators (QIs) to assess compliance with current care standards through a systematic evidence search and expert consensus. Predefined QIs have been estimated for 22 EUSOMA-certified breast centers—including in Portugal—from 2006 to 2015, with minimum standards of care achieved in 8 of the 13 main Qis in 2006 and in all Qis in 2015. Compliance with guidelines, reflected by better performance in Qis, significantly improved over the years in EUSOMA-certified breast centers [3,4]. These indicators may be useful for identifying gaps and areas for quality improvement at local and national levels [5].

The 360 Health Analysis (H360) project is a pioneering multiphase project that aims to provide a comprehensive picture of breast cancer management in Portugal by retrieving real-world data from Portuguese hospitals. After a project presentation and a literature review of the subject in H360 Phase 1, Phase 2 aimed to assess the performance of the Portuguese health system in breast cancer management by comparing the realities of different hospitals and cancer centers in the country. H360 Phase 2 is comprised of two stages: Stage A retrieves secondary data from patients’ clinical records for measuring KPIs in breast cancer, and Stage B retrieves primary data from patients, healthcare professionals, and hospital decision-makers about the patient journey within the health system. The final goal is to put forward a national consensus with an action plan on how to improve breast cancer management in Portugal [6].

The present analysis concerns the H360 project Phase 2, Stage A, and focuses on the assessment of KPIs in breast cancer screening, diagnosis, treatment, staging, and follow-up. To the authors’ knowledge, this is the first study focusing on quality indicators in breast cancer management in Portugal.

## 2. Materials and Methods

This was a retrospective study conducted between February 2019 and August 2020 using a sample of Portuguese hospitals. A set of KPIs was established based on QIs previously identified by EUSOMA and performance measures set by Khare SR et al., 2016 [2,3]. Data were collected from the clinical records of patients randomly assigned to each participating hospital until the predefined sample size for each hospital was achieved. 

Study inclusion criteria comprised women with ≥18 years of age and a diagnosis of breast cancer corresponding to a first cancer diagnosis and established ≥6 months and ≤5 years ago. No exclusion criteria were defined. Sampling was randomly conducted by study investigators.

The pool of selected hospitals included general hospitals and oncology centers, and public and private hospitals.

Based on a sample of 10 hospitals and a 1.72% prevalence of breast cancer in western Europe [7], the initially estimated sample size ranged between 263 and 332 patients. Considering a bilateral test with a 0.05 probability of type I error and 0.95 potency, the estimated sample size using G*Power^®^ software version 3.1.9.3 was 300 patients.

Data descriptive analysis was performed through absolute and relative frequencies (qualitative variables) or mean and standard deviation (quantitative variables).

KPI compliance with minimum and target standards defined by EUSOMA in each disease stage was statistically assessed based on the proportion of patients (and 95% confidence interval [CI]) with compliance in each KPI [3]. KPI compliance according to type of hospital and disease stage was assessed using Pearson chi-square or Fisher’s exact test.

Data analysis was performed using IBM SPSS^®^ version 26, adopting a 0.05 significance level.

This study was approved by the administration boards of participating hospitals following approval by the respective ethics committees, and its design and conception were the strict responsibility of the study investigators. 

## 3. Results

Of the ten hospitals initially selected, three were excluded due to successive bureaucratic and Ethic and Data Protection Commission delays, making a final sample of seven hospitals.

The target number of QIs retrieved from clinical records in each hospital was 40. The study sample was evenly distributed among hospitals and disease stages.

- KPI compliance—Descriptive analysis

QIs were evaluated for a total of 259 patients in the seven hospitals included in the study (Table 1). Hospitals 1 and 2 were oncology centers; hospital 3 was a general university hospital; hospitals 4, 5, and 6 were general hospitals; and hospital 7 was the only private hospital in the sample. Some general hospitals were district hospitals.

The planned number of patients was met in four of the seven hospitals, with the remaining hospitals achieving 58–93% of the accrual target. Patients’ clinical records were assessed for compliance with a total of 31 KPIs (Table 2).

1. Screening

Analysis of screening KPIs showed that approximately one-third of patients (34.8%) were diagnosed through a screening exam, hence without symptoms at diagnosis.

2.  Diagnosis

Two-thirds of patients with stage I–III breast cancer and 82% of patients with metastatic breast cancer were symptomatic at the time of diagnosis. Almost all patients underwent mammography and ultrasound exams at diagnosis. Core biopsy was the method of choice for histological diagnosis in most patients (71–85% of patients, depending on the stage). Histological type, grade, hormone receptor (HR) expression, and human epidermal growth factor receptor 2 (HER2) amplification were evaluated in most biopsies performed (>90%). These parameters were also assessed on the surgical specimen for most patients (77–100%), in addition to stage, size, lymphovascular invasion, and margins for invasive and in situ components. Approximately half of patients with localized disease underwent breast magnetic resonance imaging (MRI) before surgery. One-fifth of patients were referred to genetic counseling, even in metastatic settings. The time from the diagnostic biopsy to the initial breast cancer surgery or systemic treatment varied between 4 and 180 days.

3.  Treatment (stages I–III)

All patients with localized diseases had their cases discussed in multidisciplinary group meetings.

More than 90% of patients with early-stage breast cancer (stages I or II) and 68% of patients with stage III breast cancer and clinically negative axillary nodes underwent sentinel node biopsy. Regarding localized tumors, the higher the stage, the higher the percentage of patients who underwent axillary clearance (at least 10 lymph nodes). On the other hand, 83% of pN0 patients did not undergo axillary clearance.

Almost all patients underwent a single surgery (excluding reconstruction) for their primary tumor. Most patients (83%) with axillary lymph node involvement (≥pN2a) received postmastectomy radiotherapy (RT) for unresectable regional lymph nodes and the chest wall. Among patients with invasive breast cancer no larger than 3 cm in size (including the ductal carcinoma in situ [DCIS] component), 66% underwent breast-conserving surgery as a primary treatment.

Almost all patients (97%) with endocrine-sensitive invasive cancer received endocrine therapy, and 66% of patients with HR-negative (T > 1 cm or node-positive) invasive carcinoma received adjuvant chemotherapy.

Ninety-two percent of patients with HER2-positive (immunohistochemistry [IHC] 3+ or in situ hybridization-positive) invasive carcinoma (T > 1 cm or node-positive) treated with chemotherapy received adjuvant trastuzumab.

4.  Staging and follow-up

Half of patients with stage I disease did not undergo baseline staging tests during initial staging, as opposed to 90% of patients with stage III disease. Almost all patients were referred to nurse counseling at the time of the first visit or prior to the primary treatment with the purpose of being informed about day-to-day hospital functioning, assessment of body surface/surgical scars, and toxicities associated with the proposed treatment.

After curative treatment, almost all patients underwent routine annual mammography surveillance and clinical evaluation with a periodicity of 6–12 months in the first five years after the primary surgery.

- KPI compliance with target EUSOMA standards in all localized disease stages

KPIs were assessed and compared with target EUSOMA standards. KPI compliance with minimum and target EUSOMA standards in stage I–III breast cancer is detailed in Table 3. Some KPIs in this study were adapted and hence had no corresponding target standard as per the EUSOMA definition, so they were not statistically compared with QIs.

As shown in Table 3, a total of 19 diagnosis, treatment, staging, and follow-up KPIs achieved the target EUSOMA standards and six achieved the minimum EUSOMA standards, whereas 15 KPIs did not meet the minimum EUSOMA standards.

1. Diagnosis

Full compliance with EUSOMA standards was observed in women with breast cancer who preoperatively underwent mammography—99.5% (95% CI 97–100%)—and ultrasonography of both breasts and axillae—99% (95% CI 96–100%). However, minimum standards were not met for physical examination.

Full compliance with EUSOMA standards was also observed for preoperative histology- or cytology-confirmed malignant diagnosis—96% (95% CI 94–99%)—as well as for descriptive histological parameters (histological type, grade, estrogen receptor [ER], and progesterone receptor [PgR] expression, and HER2 amplification).

Regarding the pathological report of the surgical specimen, histological type and grade met target standards—99.4% (95% CI 98–100%) and 98.2% (95% CI 96–100%), respectively—while pathological stage (pT and pN, or ypT and ypN in the case of PST) only met minimum standards. All the remaining parameters failed to meet predefined EUSOMA standards. For noninvasive cancers, only the dominant histological pattern reported met the target standard—98.3% (95% CI 95–100%)—with the remaining parameters falling below minimum standards.

2.  Treatment

All stage I–III breast cancer cases were discussed in a multidisciplinary group meeting, which included surgeons, medical oncologists, pathologists, radiologists, radiation oncologists, geneticists, and nuclear medicine specialists. Considering surgical parameters, KPI compliance with standards was met for patients with invasive cancer and clinically negative axilla who underwent sentinel lymph node biopsy—90.6% (95% CI 86–96%)—and for patients with invasive breast cancer pN0 who did not undergo axillary clearance—82.9% (95% CI 76–90%). Minimum standards were met for patients with invasive cancer who received a single surgery for the primary tumor—83.9% (95% CI 78–90%)—and for patients with invasive breast cancer <3 cm in size (including DCIS component) who underwent breast-conserving treatment as primary treatment (excluding BRCA1 and BRCA2 patients)—65.7% (95% CI 58–74%). Patients with invasive cancer who underwent axillary clearance failed to meet the minimum criteria of at least 10 excised nodes.

Regarding RT, the proportion of patients with pN2a involvement who received post-mastectomy RT to the chest wall and all unresectable regional lymph nodes met target EUSOMA standards—82.9% (95% CI 70–95%). However, the proportion of patients with invasive breast cancer who received postoperative RT after surgical resection of the primary tumor and appropriate axillary staging/surgery in the setting of breast-conserving therapy fell below minimum standards.

For systemic therapy, two out of seven KPIs met target standards: proportion of patients with endocrine-sensitive invasive cancer who received endocrine therapy—97.4% (95% CI 95–100%)—and proportion of patients with HER2-positive invasive carcinoma (T > 1 cm or node-positive) treated with chemotherapy who received adjuvant trastuzumab—92% (95% CI 81–103%). Compliance with minimum standards was met for the proportion of patients with HER2-negative invasive carcinoma (T > 1 cm or node-positive) treated with chemotherapy who did not receive adjuvant trastuzumab (96.3% [95% CI 93–99%]) and for the proportion of patients with ER-negative invasive carcinoma (T > 1 cm or node-positive) who received adjuvant chemotherapy (65.6% [95% CI 49–82]). The remaining three parameters evaluating systemic therapy failed to meet minimum standards.

3.  Staging and follow-up

Regarding staging and follow-up KPIs, the target standard was 99%, and the minimum standard was 95%. Except for women with stage I breast cancer who did not undergo baseline staging tests, all three remaining KPIs met minimum standards (two of which met target standards).

- KPI compliance with target EUSOMA standards according to disease stage

KPI compliance with target EUSOMA standards according to disease stage was achieved for most KPIs. The analysis was not performed for some KPIs due to the small sample size for some disease stage parameters.

Compliance with target EUSOMA standards in stage I disease was not achieved for the following KPIs: Proportion of patients with invasive cancer who underwent axillary clearance with excision of at least 10 nodes; proportion of patients with invasive breast cancer who received postoperative RT after surgical resection of the primary tumor and appropriate axillary staging/surgery in the setting of breast-conserving treatment; and proportion of patients with stage I breast cancer who did not undergo baseline staging tests (Table 4).

For invasive and noninvasive cancers with prognostic/predictive parameters recorded in the surgical specimen, not all parameters met target standards.

In stage II disease, minimum EUSOMA standards were not achieved for the following KPIs: proportion of patients with invasive cancer with ER and PgR expression and HER2 amplification recorded in the surgical specimen, and proportion of patients with a report of peritumoral vascular invasion and distance to the nearest radial margin (Table 5). The latter also failed to achieve minimum standards in noninvasive cancer cases (together with size in mm). In addition, also the proportion of patients with invasive cancer who underwent axillary clearance with excision of at least 10 nodes, the proportion of patients without endocrine-sensitive invasive cancer who did not receive endocrine therapy, and the proportion of patients with HER2-invasive carcinoma (T > 1 cm or node-positive) treated with adjuvant chemotherapy failed to meet minimum EUSOMA standards.

In stage III disease, the following KPIs failed to meet minimum EUSOMA standards: proportion of patients with invasive cancer with peritumoral vascular invasion and distance to nearest radial margin recorded in the surgical specimen; proportion of patients with noninvasive cancer with size and grade recorded in the surgical specimen; proportion of patients with invasive cancer and clinically negative axilla who only underwent sentinel lymph node biopsy; proportion of patients with invasive cancer who underwent axillary clearance with excision of at least 10 nodes; proportion of patients with invasive breast cancer who received postoperative RT after surgical resection of the primary tumor and appropriate axillary staging/surgery in breast-conserving treatment setting; and proportion of patients (excluding BRCA1 and BRCA2 cases) with invasive breast cancer no larger than 3 cm in size (including DCIS component) who underwent breast-conserving treatment (Table 6).

- KPI compliance with target EUSOMA standards according to the type of hospital and disease stage

The analysis of KPI compliance with target EUSOMA standards according to type of hospital (general hospital vs. oncology center) and disease stage is reported in Table 7 and Table 8, respectively. Regarding the type of hospital, the most statistically significant differences between general hospitals and oncology centers were in diagnostic parameters, specifically in the lower use of core biopsy and higher reporting of grade, ER and PgR expression, HER2 amplification, pathological stage, and distance to the nearest radial margin in noninvasive cancers in general hospitals compared to oncology centers. Regarding treatment parameters, the proportion of patients (with invasive cancer only) who received a single breast surgery (excluding reconstruction) for the primary tumor was the only parameter showing differences in KPI compliance between both types of hospitals, being 90% in general hospitals compared to 71.9% in oncology centers. KPI compliance according to disease stage showed statistically significant differences in some parameters, most of which concerned treatment (Table 8).

## 4. Discussion

Although several studies have examined the quality of breast cancer care, many were conducted before 2003, when guidelines and treatments differed from those currently in use, with only a few recent studies conducted at a population level [8,9,10,11].

Assessment of the performance of the health system in breast cancer management requires measuring compliance with standards of care in real life. To our knowledge, the present multicenter study represents one of the very few in the literature evaluating QIs in breast cancer care. In 2019, three population-based studies focusing on EUSOMA QIs in breast cancer management were conducted in Slovenia, France, and Norway [5,12,13]. In these studies, most EUSOMA KPIs concerned nonmetastatic disease stages. The metastatic stage has been underrepresented in studies and, due to methodological limitations, could not be properly assessed in the present study. The results of those studies are difficult to extrapolate to Portuguese reality due to differences in health systems among countries. For instance, the Portuguese health system has some distinguishing features, such as the relevance of the public sector (which is accessible and free of charge for all patients with cancer), the provider’s freedom of choice in treatment selection, and no limitation in the use of services.

The seven hospitals included in this study had distinct typologies (general hospitals/oncology centers, central/regional hospitals, public/private hospitals) and hence varied in organizational features and the number of patients with breast cancer annually admitted. The EUSOMA criteria for QIs are precise in their specifications and data selection regarding minimum and target standards [3]. These standards should be met regardless of hospital differences. The present study evaluated compliance with QIs, seeking to compare oncology centers and general hospitals as the two main hospital typologies.

The QIs selected in this study referred to screening, diagnosis, treatment, and follow-up. KPIs were mostly extracted and adapted from EUSOMA guidelines, but also from a study by Khare and colleagues addressing important QIs, including the proportion of patients with breast cancer discussed in multidisciplinary tumor boards at some point after diagnosis (as opposed to the stricter EUSOMA definition that restricts multidisciplinary boards to pre- and postoperative settings) [2,3,14]. In addition, following population-based studies from other countries, screening KPIs were included in this study in an effort to understand how patients are diagnosed while being asymptomatic [5,11,13].

Some QIs were further divided into several KPIs, with the aim of retrieving more detailed information on how breast cancer care is provided in the hospitals included. For example, the “proportion of women with breast cancer who preoperatively underwent mammography, physical examination, and ultrasound of both breasts and axillae” was subdivided into three independent KPIs focusing on mammography, physical examination, and ultrasound.

Overall, some unexpected and interesting results were found when analyzing the data retrieved from this study.

### 4.1. Hospital Performance in Breast Cancer Screening and Diagnosis

Results from this study showed that approximately one-third of women were diagnosed through screening exams. However, it should be noted that data were collected regardless of patients’ age (in Portugal, screening is indicated between the ages of 50 and 69 years), and hence a lower percentage than expected was retrieved.

Overall, the proportion of invasive cancer cases for which the histological type, grade, ER and PgR expression, and HER2 amplification were reported was over 95%, which agrees with EUSOMA target standards. However, this was not observed for the surgical specimen. We believe this is in part because it is not mandatory to specifically repeat ER/PgR expression and HER2 amplification in the surgical piece for patients with previous assessment of these parameters in the biopsy. Pathological in situ data also failed to meet EUSOMA standards. However, while some parameters were considered risk factors in the past (such as distance to the nearest radial margin), reference to a free tumor margin is currently considered sufficient.

The proportion of cancer cases preoperatively examined by MRI was considerably higher than EUSOMA target standards. However, we hypothesize that this proportion is overestimated, as all patients were included regardless of the therapeutic strategy (namely, women receiving neoadjuvant systemic therapy). Additionally, approximately one-fifth of patients were referred for genetic counseling, when the EUSOMA target is 5%.

### 4.2. Hospital Performance in Breast Cancer Treatment

EUSOMA standards for surgical procedures were globally met. The EUSOMA consensus was based on a meta-analysis, including 28,162 patients from 33 studies examining the relationship between margin width and local control [15]. Two-thirds of the EUSOMA working group recommended an average reoperation rate of less than 20% in 2020 [3]. The proportion of patients with invasive cancer who received a single surgery (excluding reconstruction) for the primary tumor was similar to that reported in most European studies, with re-excision rates of approximately 16% [9,10,16]. American studies reported higher rates (25–40%), and a Norwegian study reported a lower re-excision rate of 6% [13,17]. However, a significant difference was found between general hospitals and oncology centers regarding distance to the nearest radial margin in noninvasive cancers. When evaluating the axillary approach, the proportion of patients with invasive cancer and a clinically negative axilla who underwent sentinel lymph node biopsy was above 90%, complying with target EUSOMA standards and representing a higher proportion than in several European studies [5,12]. The proportion of patients with invasive breast cancer pN0 who did not undergo axillary clearance also met target EUSOMA standards. However, when evaluating patients with invasive cancer who underwent axillary clearance with excision of at least 10 nodes, the results of this study fell short of target EUSOMA standards [3,14]. Future studies should address this issue to investigate whether it is a national or regional issue, and strategies directed at improving QIs should be implemented.

The proportion of patients with invasive breast cancer who received postoperative RT after surgical resection in the setting of breast-conserving therapy was below minimum EUSOMA standards, diverging from European studies, where higher rates (92–98%) were reported [5,12,13]. In the US, RT after breast-conserving therapy is less frequent (80%) and shows geographic disparities [18,19]. These disparities should also be addressed in future studies, including in Portugal, to investigate whether they represent a national issue or are hospital specific. Acknowledging this unmet QI can be a starting point for the implementation of strategies by health professionals and hospital administrations.

Concerning antineoplastic endocrine therapy, the results here obtained agree with those from previous studies [5,9,10,12,16,20] and indicate a lack of compliance with guidelines, which recommend endocrine therapy for all endocrine-positive breast cancers except small tumors (pT1aN0) [21]. We believe that this low compliance may be related to patient-related factors or tumor characteristics (very early-stage disease, weak HR positivity) and to the harm/benefit ratio. On the other hand, the proportion of patients without endocrine-sensitive invasive cancer who did not receive endocrine therapy failed to meet target EUSOMA standards. The interpretation of this result is not straightforward due to the low number of patients with ER- and PgR-negative histology included in this study.

Compliance with adjuvant chemotherapy recommendations was generally low, which can be partly explained by the use of neoadjuvant chemotherapy, which has been largely implemented in recent years, particularly in breast tumors with aggressive phenotypes (triple-negative or HER2-positive). This hypothesis is reinforced by the low number of patients eligible for KPI assessment. Furthermore, it also explains the low proportion of patients with HER2-positive disease treated with adjuvant chemotherapy. Compliance with adjuvant trastuzumab was high in this study (92%), as expected.

The proportion of patients with inflammatory or locally advanced unresectable ER-positive carcinoma who received neoadjuvant chemotherapy was lower than expected.

Although, in general, small differences were found in KPI compliance among institutions, several statistical differences were found in treatment KPIs according to disease stage. This is probably related to the variability of treatment options available for each breast cancer subtype, particularly in stage III disease, where treatment guidelines are less specific than in other disease stages and the variability of treatment options is greater.

### 4.3. Hospital Performance in Breast Cancer Staging and Follow-Up

Due to the exceedingly low probability (0.3–1.2%) of distant occult metastases in stages I–II [3], the current guidelines recommend imaging assessment only for the staging of patients with symptomatic early and stage III breast cancer [21]. In the present study, compliance with target EUSOMA standards in stage III disease staging was very high, contrary to what happened in stages I–II. However, the fact that it was not possible to distinguish between asymptomatic and symptomatic stage I disease may hamper data interpretation. This lack of compliance with target standards in early disease may also be partially explained by patient- and physician-related factors, namely patient-driven physician behavior of ordering unnecessary tests and fear of malpractice litigation. Given the costs and morbidity associated with unnecessary tests, an investment should be made in educating both patients and physicians to perform only the necessary tests, for instance through educational sessions and initiatives promoting the discussion and dissemination of guidelines about the tests and exams required for breast cancer diagnosis and staging. In addition, deciding on which tests and exams to perform for each patient in the setting of a multidisciplinary meeting may also reduce the burden of unnecessary tests.

Regarding follow-up, a recent study indicated that although not all Portuguese institutions treating patients with breast cancer have an established written protocol for follow-up of breast cancer survivors, most follow and comply with international guidelines for that purpose [22], which justifies the consistency in the results obtained among different institutions in the present study.

The main differences in KPI compliance between general hospitals and oncology centers concerned diagnosis. These included, for instance, lower use of core biopsy and higher reporting of tumor grade, ER/PgR expression, and HER2 amplification, pathological stage, and distance to the nearest radial margin in noninvasive cancers in general hospitals compared to oncology centers. Treatment KPIs were generally similar between both hospital typologies, except for the proportion of patients with invasive cancer who received a single breast surgery (excluding reconstruction) for the primary tumor, which showed higher compliance with standards in general hospitals, reaching the target standard defined by EUSOMA.

Differences in KPI compliance among hospitals may be related not only to patient and tumor characteristics but also to hospital and physician-related features. Indeed, diagnostic and surgical procedures may vary according to clinical practice and organizational factors. Regional differences partly determine differences in the type of hospital present in the region and in the provision of health care between hospitals, namely regarding screening, access to some treatments [e.g., RT], and coordination of care. Patient characteristics and physician preferences may also partly explain some of the differences observed. However, compliance with quality standards of care should be met regardless of the number of patients referred to each hospital, the type of hospital, or the geographic region. In this study, differences in KPI compliance according to disease stage mainly concerned treatment, as different stages are treated differently. Additionally, as expected, most asymptomatic patients were associated with earlier disease stages.

### 4.4. Study Limitations

This study has limitations that should be acknowledged. Its retrospective nature limited data collection to what is documented in patients’ clinical records. It would also have been desirable to have a higher number of clinical records included in the study to allow the analysis of disease stages according to individual hospitals, on the one hand, and enable comparisons between hospitals, on the other. The fact that only one private hospital was included also represents a study limitation, as it hampered the analysis of breast cancer management in the private sector. Consequently, it can be argued that results from this study should be adjusted for case mix (differences in patient populations) to validate comparisons. The fact that it was not possible to retrieve information on hereditary breast cancer or exclude these cases from the analysis may have had an impact on the study findings (although it is unlikely to be significant).

The analysis of patients with metastatic cancer was another study limitation, as some were stage IV patients and others were primarily locally treated, later progressing to metastatic disease. The heterogeneity of this subgroup in the study hampered the interpretation of the results and the analysis of several treatment parameters. In Portugal, each institution provides its own palliative care and support for these patients, which always includes psychological and/or social support throughout the course of the disease. Some institutions provide this support from the moment of metastatic disease diagnosis, and others after the conclusion of targeted cancer therapy.

Future studies with a larger sample size addressing the asymmetries reported in this study are required, as they may provide additional insights into which KPIs should be extensively explored and which measures should be implemented to improve KPIs by disease stage and hospital. The implementation of KPI measures on the National Oncology Registry (Registo Oncológico Nacional [RON]) is of particular interest to retrieve a national picture of breast cancer management. The inclusion of a comprehensive patient population would enable measuring outcomes and their variation between hospitals according to compliance with QIs.

Finally, future research directions should also be highlighted, such as research focusing on patient-centered care, shared decision-making, and personalized oncology.

The findings of this study and their implications should be discussed in light of health policies in breast cancer management in Portugal.

## 5. Conclusions

The relevance of monitoring the performance of breast-treating centers in disease management through a set of QIs is largely demonstrated by numerous initiatives undertaken at the national and international levels. EUSOMA QIs are a crucial aspect of the voluntary European certification process, based on EUSOMA ‘Requirements of a Specialist Breast Centre’.

The present study provides the first comprehensive overview of the quality of breast cancer care at a national level in Portugal. Globally, QIs meet EUSOMA standards for the diagnosis and treatment of nonmetastatic breast cancer, but there is room for improvement. Regarding treatment, stage III disease shows the greatest variability in KPI compliance with EUSOMA standards, probably due to the low specificity of the guidelines at this stage. Due to methodological limitations, no conclusions can be drawn regarding metastatic disease.

Relevant insights were retrieved on how breast cancer care in Portugal compares to European standards, which can be used as a starting point for further studies on how to improve the clinical practice of breast cancer management and update national guidelines. Clinical practice guidelines inform evidence-based cancer management, and their consistent adoption is crucial to standardizing the quality of cancer care. Implementing KPI assessment in the National Oncologic Registry (RON) can also provide more extensive and detailed data on the quality of breast cancer care nationally.

## Figures and Tables

**Table 1:** Number of patients included according to hospital and disease stage.

Hospitals	Stage I	Stage II	Stage III	Stage IV
Hospital 1	10	10	10	10
Hospital 2	10	9	7	10
Hospital 3	10	10	10	10
Hospital 4	10	11	10	10
Hospital 5	10	10	10	7
Hospital 6	10	10	10	11
Hospital 7	2	11	0	10

**Table 2:** KPI compliance according to disease stage.

KPI										
	Stage I, II, and III								Metastatic Setting	
	Total (Stage I, II, and III)		Stage I		Stage II		Stage III			
	N	n (%)	N	n (%)	N	n (%)	N	n (%)	N	n (%)
Screening and diagnosis										
1. With signs or symptoms (%)	186	121 (65.1)	60	28 (46.7)	70	44 (62.9)	56	49 (87.5)	67	55 (82.1)
2. Proportion of women with breast cancer who preoperatively underwent: (%)										
2.1. Mammography	191	190 (99.5)	62	62 (100)	72	72 (100)	57	56 (98.2)	68	66 (97.1)
2.2. Physical examination	191	157 (82.2)	62	50 (80.6)	72	60 (83.3)	57	47 (82.5)	67	55 (82.1)
2.3. Ultrasound of both breasts and axillae	191	189 (99.0)	62	61 (98.4)	72	72 (100)	57	56 (98.2)	68	66 (97.1)
3. Anatomopathological diagnosis (%)										
3.1. Fine-needle aspiration	191	48 (25.1)	62	16 (25.8)	72	13 (18.1)	57	19 (33.3)	66	21 (31.8)
3.2. Core-needle biopsy	191	149 (78.0)	62	44 (71.0)	72	61 (84.7)	57	44 (77.2)	68	58 (85.3)
3.3. Surgical biopsy	191	35 (18.3)	62	13 (21.0)	72	10 (13.9)	57	12 (21.1)	66	2 (3.0)
4. Proportion of women with breast cancer (invasive or in situ) with preoperative histologically or cytologically confirmed malignant diagnosis (%)	189	182 (96.3)	62	57 (91.9)	71	70 (98.6)	56	55 (98.2)	-	-
5. Proportion of invasive cancer cases for which the following prognostic/predictive parameters were recorded: (%)										
5.1. Histological type	184	178 (96.7)	58	54 (93.1)	70	69 (98.6)	56	55 (98.2)	66	66 (100)
5.2. Grade	183	175 (95.6)	58	52 (89.7)	69	68 (98.6)	56	55 (98.2)	66	65 (98.5)
5.3. ER and PgR expression	183	173 (94.5)	58	53 (91.4)	69	66 (95.7)	56	54 (96.4)	66	65 (98.5)
5.4. HER2 amplification	182	171 (94.0)	58	53 (91.4)	69	64 (92.8)	55	54 (98.2)	66	65 (98.5)
6. Proportion of invasive cancer cases for which the following prognostic/predictive parameters were recorded in the surgical specimen: (%)										
6.1. Histological type	172	171 (99.4)	61	61 (100)	67	66 (98.5)	44	44 (100)	-	-
6.2. Grade	167	164 (98.2)	60	59 (98.3)	66	66 (100)	41	39 (95.1)	-	-
6.3. ER and PgR expression	170	143 (84.1)	61	47 (77.0)	66	57 (86.4)	43	39 (90.7)	-	-
6.4. HER2 amplification	169	142 (84.0)	61	47 (77.0)	66	56 (84.8)	42	39 (92.9)	-	-
6.5. Pathological stage (pT and pN, or ypT and ypN in case of PST)	170	157 (92.4)	61	57 (93.4)	67	60 (89.6)	42	40 (95.2)	-	-
6.6. Size in mm of the invasive component	169	151 (89.3)	61	55 (90.2)	66	60 (90.9)	42	36 (85.7)	-	-
6.7. Peritumoral vascular invasion	167	144 (86.2)	60	55 (91.7)	65	54 (83.1)	42	35 (83.3)	-	-
6.8. Distance to the nearest radial margin	161	143 (88.8)	58	56 (96.6)	63	54 (85.7)	40	33 (82.5)	-	-
7. Proportion of noninvasive cancer cases for which the following prognostic/predictive parameters were recorded: (%)									-	
7.1. Dominant histological pattern	59	58 (98.3)	24	23 (95.8)	23	23 (100)	12	12 (100)	-	-
7.2. Size in mm	59	41 (69.5)	24	18 (75.0)	23	15 (65.2)	12	8 (66.7)	-	-
7.3. Grade	59	46 (78.0)	24	20 (83.3)	23	19 (82.6)	12	7 (58.3)	-	-
7.4. Distance to the nearest radial margin	57	36 (63.2)	23	13 (56.5)	22	13 (59.1)	12	10 (83.3)	-	-
8. Time interval of 6 weeks from the date of the first diagnostic examination in the breast center to the date of surgery or treatment start	181	57.7 (35.4) 4–180	58	67.5 (35.0) 14–180	70	54.4 (36.0) 7–153	53	51.2 (33.2) 4–175	59	48.9 (27.8) 9–121
9. Proportion of cancer cases preoperatively examined by MRI (%)	188	99 (52.7)	61	20 (32.8)	71	41 (57.7)	56	38 (67.9)	-	-
10. Proportion of cancer cases referred to genetic counseling (%)	181	39 (21.5)	62	8 (12.9)	67	16 (23.9)	52	15 (28.8)	59	12 (20.3)
Treatment										
1. Proportion of cancer patient cases discussed in multidisciplinary group meeting (%)	190	190 (100)	61	61 (100)	72	72 (100)	57	57 (100)	68	67 (98.5)
2. Proportion of patients (invasive cancer only) who received a single (breast) surgery for the primary tumor (excluding reconstruction) (%)	161	135 (83.9)	52	45 (86.5)	64	52 (81.3)	45	38 (84.4)	-	-
3. Proportion of patients with invasive cancer and clinically negative axilla who only underwent SLNB (%)	128	116 (90.6)	54	52 (96.3)	55	51 (92.7)	19	13 (68.4)	-	-
4. Proportion of patients with invasive cancer who underwent axillary clearance with excision of at least 10 nodes	130	59 (45.4)	33	6 (18.2)	47	17 (36.2)	50	36 (72.0)	-	-
5. Proportion of patients with invasive breast cancer (M0) who received postoperative RT after surgical resection of the primary tumor and appropriate axillary staging/surgery in BCT setting (%)	149	117 (78.5)	57	48 (84.2)	58	49 (84.5)	34	20 (58.8)	-	-
6. Proportion of patients with axillary lymph node involvement (pN2a) who received postmastectomy RT to the chest wall and all (nonresected) regional lymph-nodes (%)	35	29 (82.9)	1	0 (0.0)	6	4 (66.7)	28	25 (89.3)	-	-
7. Proportion of patients (excluding BRCA1 and BRCA2 patients) with invasive breast cancer no larger than 3 cm (total size, including DCIS component) who underwent BCT as a primary treatment (%)	140	92 (65.7)	58	47 (81.0)	57	35 (61.4)	25	10 (40.0)	-	-
8. Proportion of patients with invasive breast cancer who underwent axillary clearance (%)	182	71 (39.0)	61	7 (11.5)	69	23 (33.3)	52	41 (78.8)	-	-
9. Proportion of patients with invasive breast cancer pN0 who did not undergo axillary clearance (%)	105	87 (82.9)	54	47 (87.0)	36	31 (86.1)	15	9 (60.0)	-	-
10. Proportion of patients with endocrine-sensitive invasive cancer who received endocrine therapy (%)	155	151 (97.4)	57	54 (94.7)	58	57 (98.3)	40	40 (100)	-	-
11. Proportion of patients without endocrine-sensitive invasive cancer who did not receive endocrine therapy (%)	35	30 (85.7)	5	4 (80.0)	15	11 (73.3)	15	15 (100)	-	-
12. Proportion of patients with ER-negative (T > 1 cm or node +) invasive carcinoma who received adjuvant chemotherapy (%)	32	21 (65.6)	5	4 (80.0)	14	8 (57.1)	13	9 (69.2)	-	-
13. Proportion of patients with HER2-positive (IHC 3+ or in situ hybridization-positive) invasive carcinoma (T > 1 cm or N+) treated with chemotherapy who received adjuvant trastuzumab (%)	25	23 (92.0)	6	5 (83.3)	10	9 (90.0)	9	9 (100)	-	-
14. Proportion of patients with HER2-negative invasive carcinoma (T > 1 cm or N+) treated with chemotherapy who did not receive adjuvant trastuzumab (%)	160	154 (96.3)	53	51 (96.2)	61	58 (95.1)	46	45 (97.8)	-	-
15. Proportion of patients with HER2-positive (IHC 3+ or in situ hybridization positive) invasive carcinoma (T > 1 cm or N+) treated with adjuvant chemotherapy (%)	27	14 (51.9)	6	4 (66.7)	11	6 (54.5)	10	4 (40.0)	-	-
16. Proportion of patients with inflammatory breast cancer or locally advanced unresectable ER-negative carcinoma who received neoadjuvant chemotherapy (%)	68	46 (67.6)	5	0 (0.0)	15	12 (80.0)	48	34 (70.8)	-	-
Staging and follow-up										
1. Proportion of women with stage I breast cancer who did not undergo baseline staging tests (liver US, chest X-ray, and bone scan) (%)	60	32 (53.3)	60	32 (53.3)	-	-	-	-	-	-
2. Proportion of women with stage III breast cancer who underwent baseline staging tests (liver US, chest X-ray, and bone scan) (%)	55	50 (90.9)	-	-	-	-	55	50 (90.9)	-	-
3. Proportion of asymptomatic patients who underwent routine annual mammography screening and 6/12-month clinical evaluation in the first 5 years after primary surgery (%)	173	169 (97.7)	60	60 (100)	69	66 (95.7)	44	43 (97.7)	-	-
4. Proportion of women with breast cancer diagnosis with direct access to a breast care nurse specialist for information and support regarding treatment-related symptoms and toxicity, follow-up, and rehabilitation after initial treatment (%)	189	187 (98.9)	61	59 (96.7)	72	72 (100)	56	56 (100)	67	66 (98.5)

BCT, breast-conserving treatment; DCIS, ductal carcinoma in situ; ER, estrogen receptor; HER2, human epidermal growth factor receptor 2; IHC, immunohistochemistry; KPI, key performance indicator; MRI, magnetic resonance imaging; n, number of patients with compliance with the respective KPI; N, number of patients evaluated for the KPI; PgR, progesterone receptor; PST, primary systemic treatment; RT, radiotherapy; SLNB, sentinel lymph node biopsy; US, ultrasonography.

**Table 3:** KPI compliance with minimum and target EUSOMA standards in stage I–III breast cancer.

	Total (Stage I, II, and III)			EUSOMA Minimum Target	Results
	N	n (%)	95% CI		
Screening and diagnosis					
1. With signs or symptoms (%)	186	121 (65.1)	-	-	
2. Proportion of women with breast cancer who preoperatively underwent: (%)					
2.1. Mammography	191	190 (99.5)	0.971–1.000	90–95%	✓✓
2.2. Physical examination	191	157 (82.2)	0.760–0.873	90–95%	✗
2.3. Ultrasound of both breasts and axillae	191	189 (99.0)	0.963–0.999	90–95%	✓✓
3. Anatomopathological diagnosis (%)					
3.1. Fine-needle aspiration	191	48 (25.1)	-	-	
3.2. Core-needle biopsy	191	149 (78.0)	-	-	
3.3. Surgical biopsy	191	35 (18.3)	-	-	
4. Proportion of women with breast cancer (invasive or in situ) who had a preoperative histologically or cytologically confirmed malignant diagnosis (%)	189	182 (96.3)	0.936–0.990	80–90%	✓✓
5. Proportion of invasive cancer cases for which the following prognostic/predictive parameters were recorded: (%)					
5.1. Histological type	184	178 (96.7)	0.941–0.993	90–95%	✓✓
5.2. Grade	183	175 (95.6)	0.926–0.986	90–95%	✓✓
5.3. ER and PgR expression	183	173 (94.5)	0.912–0.978	90–95%	✓✓
5.4. HER2 amplification	182	171 (94.0)	0.905–0.975	90–95%	✓✓
6. Proportion of invasive cancer cases for which the following prognostic/predictive parameters were recorded in the surgical specimen: (%)					
6.1. Histological type	172	171 (99.4)	0.982–1.006	95–98%	✓✓
6.2. Grade	167	164 (98.2)	0.962–1.002	95–98%	✓✓
6.3. ER and PgR expression	170	143 (84.1)	0.786–0.896	95–98%	✗
6.4. HER2 amplification	169	142 (84.0)	0.785–0.895	95–98%	✗
6.5. Pathological stage (pT and pN, or ypT and ypN in case of PST)	170	157 (92.4)	0.884–0.964	95–98%	✓
6.6. Size in mm of the invasive component	169	151 (89.3)	0.846–0.940	95–98%	✗
6.7. Peritumoral vascular invasion	167	144 (86.2)	0.810–0.914	95–98%	✗
6.8. Distance to the nearest radial margin	161	143 (88.8)	0.839–0.937	95–98%	✗
7. Proportion of noninvasive cancer cases for which the following prognostic/predictive parameters were recorded: (%)					
7.1. Dominant histological pattern	59	58 (98.3)	0.950–1.016	95–98%	✓✓
7.2. Size in mm	59	41 (69.5)	0.578–0.812	95–98%	✗
7.3. Grade	59	46 (78.0)	0.674–0.886	95–98%	✗
7.4. Distance to the nearest radial margin	57	36 (63.2)	0.507–0.757	95–98%	✗
8. Time interval of 6 weeks from the date of the first diagnostic examination in the breast center to the date of surgery or start of treatment	181	57.7 (35.4) 4–180	-	-	
9. Proportion of cancer cases preoperatively examined by MRI (%)	188	99 (52.7)	0.456–0.598	5%	✓✓
10. Proportion of cancer cases referred for genetic counseling (%)	181	39 (21.5)	0.155–0.275	5%	✓✓
Treatment					
1. Proportion of cancer patient cases discussed in multidisciplinary group meeting (%)	190	190 (100)	-		
2. Proportion of patients (invasive cancer only) who received a single (breast) surgery for the primary tumor (excluding reconstruction) (%)	161	135 (83.9)	0.782–0.896	80–90%	✓
3. Proportion of patients with invasive cancer and clinically negative axilla who only underwent SLNB (%)	128	116 (90.6)	0.855–0.957	90–95%	✓✓
4. Proportion of patients with invasive cancer who underwent axillary clearance with at least 10 excised nodes	130	59 (45.4)	0.368–0.54	95–98%	✗
5. Proportion of patients with invasive breast cancer (M0) who received postoperative RT after surgical resection of the primary tumor and appropriate axillary staging/surgery in BCT settings (%)	149	117 (78.5)	0.719–0.851	90–95%	✗
6. Proportion of patients with axillary lymph node involvement (pN2a) who received postmastectomy RT to the chest wall and all (unresctable) regional lymph-nodes (%)	35	29 (82.9)	0.704–0.954	90–95%	✓✓
7. Proportion of patients (excluding BRCA1 and BRCA2 patients) with invasive breast cancer no larger than 3 cm (total size, including DCIS component) who underwent BCT as a primary treatment (%)	140	92 (65.7)	0.578–0.736	70–80%	✓
8. Proportion of patients with invasive breast cancer who underwent axillary clearance (%)	182	71 (39.0)	-		
9. Proportion of patients with invasive breast cancer pN0 who did not undergo axillary clearance (%)	105	87 (82.9)	0.757–0.901	80–90%	✓✓
10. Proportion of patients with endocrine-sensitive invasive cancer who received endocrine therapy (%)	155	151 (97.4)	0.949–0.999	80–90%	✓✓
11. Proportion of patients without endocrine-sensitive invasive cancer who did not receive endocrine therapy (%)	35	30 (85.7)	0.741–0.973	98–100%	✗
12. Proportion of patients with ER-negative (T > 1 cm or node +) invasive carcinoma who received adjuvant chemotherapy (%)	32	21 (65.6)	0.491–0.821	80–90%	✓
13. Proportion of patients with HER2-positive (IHC 3+ or in situ hybridization positive) invasive carcinoma (T > 1 cm or N+) treated with chemotherapy who received adjuvant trastuzumab (%)	25	23 (92.0)	0.814–1.026	80–90%	✓✓
14. Proportion of patients with HER2-negative invasive carcinoma (T > 1 cm or N+) treated with chemotherapy who did not receive adjuvant trastuzumab (%)	160	154 (96.3)	0.934–0.992	98–100%	✓
15. Proportion of patients with HER2-positive (IHC 3+ or in situ hybridization positive) invasive carcinoma (T > 1 cm or N+) treated with adjuvant chemotherapy (%)	27	14 (51.9)	0.331–0.707	98–100%	✗
16. Proportion of patients with IBC or locally advanced unresectable ER-negative carcinoma who received neoadjuvant chemotherapy (%)	68	46 (67.6)	0.565–0.787	90–95%	✗
Staging and follow-up					
1. Proportion of women with stage I breast cancer who did not undergo baseline staging tests (liver US, chest X-ray, and bone scan) (%)	60	32 (53.3)	0.407–0.659	95–99%	✗
2. Proportion of women with stage III breast cancer who underwent baseline staging tests (liver US, chest X-ray, and bone scan) (%)	55	50 (90.9)	0.833–0.985	95–99%	✓
3. Proportion of asymptomatic patients who underwent routine annual mammography screening and 6/12-month clinical evaluation in the first 5 years after primary surgery (%)	173	169 (97.7)	0.955–0.999	95–99%	✓✓
4. Proportion of women with breast cancer diagnosis with direct access to a breast care nurse specialist for information and support regarding treatment-related symptoms and toxicity, follow-up, and rehabilitation after initial treatment (%)	189	187 (98.9)	0.974–1.004	95–99%	✓✓

BCT, breast-conserving treatment; DCIS, ductal carcinoma in situ; ER, estrogen receptor; HER2, human epidermal growth factor receptor 2; IHC, immunohistochemistry; KPI, key performance indicator; MRI, magnetic resonance imaging; n, number of patients with compliance with the respective KPI; N, number of patients evaluated for the respective KPI; PgR, progesterone receptor; PST, primary systemic treatment; RT, radiotherapy; SLNB, sentinel lymph node biopsy; US, ultrasonography. Colors represent compliance with minimum and target standards according to EUSOMA. Proportions and 95% confidence intervals are presented. Blue (✓✓): compliance with minimum and target standards (confidence interval contains both proportions). Green (✓): compliance with minimum but not target standards (confidence interval contains only the minimum proportion). Red (✗): non-compliance with target standards (confidence interval does not contain minimum or target proportions).

**Table 4:** KPI compliance with target standards in stage I disease.

	Total		z	Min	Max	✓✗
	Stage I					
	N	n (%)				
Diagnosis						
Proportion of women with breast cancer who preoperatively underwent: (%)						
Mammography	62	62 (100)	1.96	1	1	✓✓
Physical examination	62	50 (80.6)	1.96	0.708	0.904	✓
Ultrasound of both breasts and axillae	62	61 (98.4)	1.96	0.953	1.015	✓✓
Proportion of women with breast cancer (invasive or in situ) who had a preoperative histologically or cytologically confirmed malignant diagnosis (%)	62	57 (91.9)	1.96	0.851	0.987	✓✓
Proportion of invasive cancer cases for which the following prognostic/predictive parameters were recorded: (%)						
Histological type	58	54 (93.1)	1.96	0.866	0.996	✓✓
Grade	58	52 (89.7)	1.96	0.819	0.975	✓✓
ER and PgR expression	58	53 (91.4)	1.96	0.842	0.986	✓✓
HER2 amplification	58	53 (91.4)	1.96	0.842	0.986	✓✓
Proportion of invasive cancer cases for which the following prognostic/predictive parameters were recorded in the surgical specimen: (%)						
Histological type	61	61 (100)	1.96	1	1	✓✓
Grade	60	59 (98.3)	1.96	0.95	1.016	✓✓
ER and PgR expression	61	47 (77.0)	1.96	0.673	0.881	✗
HER2 amplification	61	47 (77.0)	1.96	0.673	0.881	✗
Pathological stage (pT and pN, or ypT and ypN in case of PST)	61	57 (93.4)	1.96	0.872	0.996	✓✓
Size in mm of the invasive component	61	55 (90.2)	1.96	0.827	0.977	✓
Peritumoral vascular invasion (L, V)	60	55 (91.7)	1.96	0.847	0.987	✓✓
Distance to the nearest radial margin	58	56 (96.6)	1.96	0.919	1.013	✓✓
Proportion of noninvasive cancer cases for which the following prognostic/predictive parameters were recorded: (%)						
Dominant histological pattern	24	23 (95.8)	1.96	0.878	1.038	✓
Size in mm	24	18 (75.0)	1.96	0.577	0.923	✗
Grade	24	20 (83.3)	1.96	0.684	0.982	✓✓
Distance to the nearest radial margin	23	13 (56.5)	1.96	0.362	0.768	✗
Proportion of cancer cases preoperatively examined by MRI (%)	61	20 (32.8)	1.96	0.21	0.446	✓✓
Proportion of cancer cases referred for genetic counseling (%)	62	8 (12.9)	1.96	0.046	0.212	✓✓
Treatment						
Proportion of patients (invasive cancer only) who received a single breast surgery for the primary tumor (excluding reconstruction) (%)	52	45 (86.5)	1.96	0.772	0.958	✓✓
Proportion of patients with invasive cancer and clinically negative axilla who only underwent SLNB (%)	54	52 (96.3)	1.96	0.913	1.013	✓✓
Proportion of patients with invasive cancer who underwent axillary clearance with at least 10 excised nodes	33	6 (18.2)	1.96	0.05	0.314	✗
Proportion of patients with invasive breast cancer (M0) who received postoperative RT after surgical resection of the primary tumor and appropriate axillary staging/surgery in BCT setting (%)	57	48 (84.2)	1.96	0.747	0.937	✓
Proportion of patients (excluding BRCA1 and BRCA2 patients) with invasive breast cancer no larger than 3 cm (total size, including DCIS component) who underwent BCT as primary treatment (%)	58	47 (81.0)	1.96	0.709	0.911	✓✓
Proportion of patients with invasive breast cancer pN0 who did not undergo axillary clearance (%)	54	47 (87.0)	1.96	0.78	0.96	✓✓
Proportion of patients with endocrine-sensitive invasive cancer who received endocrine therapy (%)	57	54 (94.7)	1.96	0.889	1.005	✓✓
Proportion of patients without endocrine-sensitive invasive cancer who did not receive endocrine therapy (%)	5	4 (80.0)	1.96	0.449	1.151	✓✓
Proportion of patients with ER-negative (T > 1 cm or node +) invasive carcinoma who received adjuvant chemotherapy (%)	5	4 (80.0)	1.96	0.449	1.151	✓✓
Proportion of patients with HER2-positive (IHC 3+ or in situ hybridization-positive) invasive carcinoma (T > 1 cm or N+) treated with chemotherapy who received adjuvant trastuzumab (%)	6	5 (83.3)	1.96	0.535	1.131	✓✓
Proportion of patients with HER2-negative invasive carcinoma (T > 1 cm or N+) treated with chemotherapy who did not receive adjuvant trastuzumab (%)	53	51 (96.2)	1.96	0.911	1.013	✓✓
Proportion of patients with HER2-positive (IHC 3+ or in situ hybridization-positive) invasive carcinoma (T > 1 cm or N+) treated with adjuvant chemotherapy (%)	6	4 (66.7)	1.96	0.29	1.044	✓✓
Staging and follow-up						
Proportion of women with stage I breast cancer who did not undergo baseline staging tests (liver US, chest X-ray, or bone scan) (%)	60	32 (53.3)	1.96	0.407	0.659	✗
Proportion of women with breast cancer diagnosis with direct access to a breast care nurse specialist for information and support regarding treatment-related symptoms and toxicity, follow-up, and rehabilitation after initial treatment (%)	61	59 (96.7)	1.96	0.922	1.012	✓✓

BCT, breast-conserving treatment; DCIS, ductal carcinoma in situ; ER, estrogen receptor; HER2, human epidermal growth factor receptor 2; IHC, immunohistochemistry; KPI, key performance indicator; MRI, magnetic resonance imaging; n, number of patients with compliance with the respective KPI; N, number of patients evaluated for the respective KPI; PgR, progesterone receptor; PST, primary systemic treatment; RT, radiotherapy; SLNB, sentinel lymph-node biopsy; US, ultrasonography. Colors represent compliance with minimum and target standards according to EUSOMA. Proportions and 95% confidence intervals are presented. Blue (✓✓): compliance with minimum and target standards (confidence interval contains both proportions). Green (✓): compliance with minimum but not target standards (confidence interval contains only minimum proportion). Red (✗): non-compliance with target standards (confidence interval does not contain minimum or target proportions).

**Table 5:** KPI compliance with target standards in stage II disease.

	Total		z	Min	Max	✓✗
	Stage II					
	N	n (%)				
Diagnosis						
Proportion of women with breast cancer who preoperatively underwent: (%)						
Mammography	72	72 (100)	1.96	1	1	✓✓
Physical examination	72	60 (83.3)	1.96	0.747	0.919	✓
Ultrasound of both breasts and axillae	72	72 (100)	1.96	1	1	✓✓
Proportion of women with breast cancer (invasive or in situ) who had a preoperative histologically or cytologically confirmed malignant diagnosis (%)	71	70 (98.6)	1.96	0.959	1.013	✓✓
Proportion of invasive cancer cases for which the following prognostic/predictive parameters were recorded: (%)						
Histological type	70	69 (98.6)	1.96	0.958	1.014	✓✓
Grade	69	68 (98.6)	1.96	0.958	1.014	✓✓
ER and PgR expression	69	66 (95.7)	1.96	0.909	1.005	✓✓
HER2 amplification	69	64 (92.8)	1.96	0.867	0.989	✓✓
Proportion of invasive cancer cases for which the following prognostic/predictive parameters were recorded in the surgical specimen: (%)						
Histological type	67	66 (98.5)	1.96	0.956	1.014	✓✓
Grade	66	66 (100)	1.96	1	1	✓✓
ER and PgR expression	66	57 (86.4)	1.96	0.781	0.947	✗
HER2 amplification	66	56 (84.8)	1.96	0.761	0.935	✗
Pathological stage (pT and pN, or ypT and ypN in case of PST)	67	60 (89.6)	1.96	0.823	0.969	✓
Size in mm of the invasive component	66	60 (90.9)	1.96	0.84	0.978	✓
Peritumoral vascular invasion	65	54 (83.1)	1.96	0.74	0.922	✗
Distance to nearest radial margin	63	54 (85.7)	1.96	0.771	0.943	✗
Proportion of noninvasive cancer cases for which the following prognostic/predictive parameters were recorded: (%)						
Dominant histological pattern	23	23 (100)	1.96	1	1	✓✓
Size in mm	23	15 (65.2)	1.96	0.457	0.847	✗
Grade	23	19 (82.6)	1.96	0.671	0.981	✓✓
Distance to the nearest radial margin	22	13 (59.1)	1.96	0.386	0.796	✗
Proportion of cancer cases preoperatively examined by MRI (%)	71	41 (57.7)	1.96	0.462	0.692	✓✓
Proportion of cancer cases referred for genetic counseling (%)	67	16 (23.9)	1.96	0.137	0.341	✓✓
Treatment						
Proportion of cancer patient cases discussed in multidisciplinary group meeting (%)	72	72 (100)	-	-	-	
Proportion of patients (invasive cancer only) who received a single breast surgery for the primary tumor (excluding reconstruction) (%)	64	52 (81.3)	1.96	0.717	0.909	✓✓
Proportion of patients with invasive cancer and clinically negative axilla who only underwent SLNB (%)	55	51 (92.7)	1.96	0.858	0.996	✓✓
Proportion of patients with invasive cancer who underwent axillary clearance with at least 10 excised nodes	47	17 (36.2)	1.96	0.225	0.499	✗
Proportion of patients with invasive breast cancer (M0) who received postoperative RT after surgical resection of the primary tumor and appropriate axillary staging/surgery in BCT settings (%)	58	49 (84.5)	1.96	0.752	0.938	✓
Proportion of patients (excluding BRCA1 and BRCA2 patients) with invasive breast cancer no larger than 3 cm (total size, including DCIS component) who underwent BCT as a primary treatment (%)	57	35 (61.4)	1.96	0.488	0.74	✓
Proportion of patients with invasive breast cancer pN0 who did not undergo axillary clearance (%)	36	31 (86.1)	-	-	-	
Proportion of patients with endocrine-sensitive invasive cancer who received endocrine therapy (%)	58	57 (98.3)	1.96	0.95	1.016	✓✓
Proportion of patients without endocrine-sensitive invasive cancer who did not receive endocrine therapy (%)	15	11 (73.3)	1.96	0.509	0.957	
Proportion of patients with ER-negative (T > 1 cm or node +) invasive carcinoma who received adjuvant chemotherapy (%)	14	8 (57.1)	1.96	0.312	0.83	✓
Proportion of patients with HER2-positive (IHC 3+ or in situ hybridization positive) invasive carcinoma (T > 1 cm or N+) treated with chemotherapy who received adjuvant trastuzumab (%)	10	9 (90.0)	1.96	0.714	1.086	✓✓
Proportion of patients with HER2-negative invasive carcinoma (T > 1 cm or N+) treated with chemotherapy who did not receive adjuvant trastuzumab (%)	61	58 (95.1)	1.96	0.897	1.005	✓✓
Proportion of patients with HER2-positive (IHC 3+ or in situ hybridization positive) invasive carcinoma (T > 1 cm or N+) treated with adjuvant chemotherapy (%)	11	6 (54.5)	1.96	0.251	0.839	✗
Proportion of patients with IBC or locally advanced unresectable ER-positive carcinoma who received neoadjuvant chemotherapy (%)	15	12 (80.0)	1.96	0.598	1.002	✓✓
Staging and follow-up						
Proportion of asymptomatic patients who underwent routine annual mammography screening and 6/12-month clinical evaluation in the first 5 years after primary surgery (%)	69	66 (95.7)	1.96	0.909	1.005	✓✓
Proportion of women with breast cancer diagnosis with direct access to a breast care nurse specialist for information and support regarding treatment-related symptoms and toxicity, follow-up, and rehabilitation after initial treatment (%)	72	72 (100)	1.96	1	1	✓✓

BCT, breast conserving treatment; DCIS, ductal carcinoma in situ; ER, estrogen receptor; HER2, human epidermal growth factor receptor 2; IHC, immunohistochemistry; KPI, key performance indicator; MRI, magnetic resonance imaging; n, number of patients with compliance with the respective KPI; N, number of patients evaluated for the respective KPI; PgR, progesterone receptor; PST, primary systemic treatment; RT, radiotherapy; SLNB, sentinel lymph node biopsy; US, ultrasonography. Colors represent compliance with minimum and target standards according to EUSOMA. Proportions and 95% confidence intervals are presented. Blue (✓✓): compliance with minimum and target standards (confidence interval contains both proportions). Green (✓): compliance with minimum but not target standards (confidence interval contains only the minimum proportion). Red (✗): non-compliance with target standards (confidence interval does not contain minimum or target proportions).

**Table 6:** KPI compliance with target standards in stage III disease.

	Total		z	Min	Max	✓✗
	Stage III					
	N	n (%)				
Diagnosis						
With signs or symptoms (%)	56	49 (87.5)	-	-	-	-
Proportion of women with breast cancer who preoperatively underwent: (%)						
Mammography	57	56 (98.2)	1.96	0.947	1.017	✓✓
Physical examination	57	47 (82.5)	1.96	0.726	0.924	✓
Ultrasound of both breasts and axillae	57	56 (98.2)	1.96	0.947	1.017	✓✓
Proportion of women with breast cancer (invasive or in situ) who had a preoperative histologically or cytologically confirmed malignant diagnosis (%)	56	55 (98.2)	1.96	0.947	1.017	✓✓
Proportion of invasive cancer cases for which the following prognostic/predictive parameters were recorded: (%)						
Histological type	56	55 (98.2)	1.96	0.947	1.017	✓✓
Grade	56	55 (98.2)	1.96	0.947	1.017	✓✓
ER and PgR expression	56	54 (96.4)	1.96	0.915	1.013	✓✓
HER2 amplification	55	54 (98.2)	1.96	0.947	1.017	✓✓
Proportion of invasive cancer cases for which the following prognostic/predictive parameters were recorded in the surgical specimen: (%)						
Histological type	44	44 (100)	1.96	1	1	✓✓
Grade	41	39 (95.1)	1.96	0.885	1.017	✓✓
ER and PgR expression	43	39 (90.7)	1.96	0.82	0.994	✓✓
HER2 amplification	42	39 (92.9)	1.96	0.851	1.007	✓✓
Pathological stage (pT and pN, or ypT and ypN in case of PST)	42	40 (95.2)	1.96	0.887	1.017	✓✓
Size in mm of the invasive component	42	36 (85.7)	1.96	0.751	0.963	✓
Peritumoral vascular invasion (L, V)	42	35 (83.3)	1.96	0.72	0.946	✗
Distance to nearest radial margin	40	33 (82.5)	1.96	0.707	0.943	✗
Proportion of noninvasive cancer cases for which the following prognostic/predictive parameters were recorded: (%)						
Dominant histological pattern	12	12 (100)	1.96	1	1	✓✓
Size in mm	12	8 (66.7)	1.96	0.4	0.934	✗
Grade	12	7 (58.3)	1.96	0.304	0.862	✗
Distance to the nearest radial margin	12	10 (83.3)	1.96	0.622	1.044	✓✓
Proportion of cancer cases preoperatively examined by MRI (%)	56	38 (67.9)	1.96	0.557	0.801	✓✓
Proportion of cancer cases referred for genetic counseling (%)	52	15 (28.8)	1.96	0.165	0.411	✓✓
Treatment						
Proportion of cancer patient cases discussed in multidisciplinary group meeting (%)	57	57 (100)	1.96	1	1	✓✓
Proportion of patients (invasive cancer only) who received a single breast surgery for the primary tumor (excluding reconstruction) (%)	45	38 (84.4)	1.96	0.738	0.95	✓✓
Proportion of patients with invasive cancer and clinically negative axilla who underwent only SLNB (%)	19	13 (68.4)	1.96	0.475	0.893	✗
Proportion of patients with invasive cancer who underwent axillary clearance with at least 10 excised nodes	50	36 (72.0)	1.96	0.596	0.844	✗
Proportion of patients with invasive breast cancer (M0) who received postoperative RT after surgical resection of the primary tumor and appropriate axillary staging/surgery in BCT settings (%)	34	20 (58.8)	1.96	0.423	0.753	✗
Proportion of patients with axillary lymph node involvement (pN2a) who received postmastectomy radiation therapy to the chest wall and all (unresectable) regional lymph nodes (%)	28	25 (89.3)	1.96	0.779	1.007	✓✓
Proportion of patients (excluding BRCA1 and BRCA2 patients) with invasive breast cancer no larger than 3 cm (total size, including DCIS component) who underwent BCT as a primary treatment (%)	25	10 (40.0)	1.96	0.208	0.592	✗
Proportion of patients with invasive breast cancer who underwent axillary clearance (%)	52	41 (78.8)	-	-	-	-
Proportion of patients with invasive breast cancer pN0 who did not undergo axillary clearance (%)	15	9 (60.0)	1.96	0.352	0.848	✓
Proportion of patients with endocrine-sensitive invasive cancer who received endocrine therapy (%)	40	40 (100)	1.96	1	1	✓✓
Proportion of patients without endocrine-sensitive invasive cancer who did not receive endocrine therapy (%)	15	15 (100)	1.96	1	1	✓✓
Proportion of patients with ER-negative (T > 1 cm or node +) invasive carcinoma who received adjuvant chemotherapy (%)	13	9 (69.2)	1.96	0.441	0.943	✓✓
Proportion of patients with HER2-positive (IHC 3+ or in situ hybridization-positive) invasive carcinoma (T > 1 cm or N+) treated with chemotherapy who received adjuvant trastuzumab (%)	9	9 (100)	1.96	1	1	✓✓
Proportion of patients with HER2-negative invasive carcinoma (T > 1 cm or N+) treated with chemotherapy who did not receive adjuvant trastuzumab (%)	46	45 (97.8)	1.96	0.936	1.02	✓✓
Proportion of patients with HER2-positive (IHC 3+ or in situ hybridization-positive) invasive carcinoma (T > 1 cm or N+) treated with adjuvant chemotherapy (%)	10	4 (40.0)	1.96	0.096	0.704	✗
Proportion of patients with IBC or locally advanced unresectable ER-positive carcinoma who received neoadjuvant chemotherapy (%)	48	34 (70.8)	1.96	0.579	0.837	✗
Staging and follow-up						
Proportion of women with stage III breast cancer who underwent baseline staging tests (liver US, chest X-ray, or bone scan) (%)	55	50 (90.9)	1.96	0.833	0.985	✓✓
Proportion of asymptomatic patients who underwent routine annual mammography screening and 6/12-month clinical evaluation in the first 5 years after primary surgery (%)	44	43 (97.7)	1.96	0.933	1.021	✓✓
Proportion of women with breast cancer diagnosis with direct access to a breast care nurse specialist for information and support regarding treatment-related symptoms and toxicity, follow-up, and rehabilitation after initial treatment (%)	56	56 (100)	1.96	1	1	✓✓

BCT, breast-conserving treatment; DCIS, ductal carcinoma in situ; ER, estrogen receptor; HER2, human epidermal growth factor receptor 2; IHC, immunohistochemistry; KPI, key performance indicator; MRI, magnetic resonance imaging; n, number of patients with compliance with the respective KPI; N, number of patients evaluated for the respective KPI; PgR, progesterone receptor; PST, primary systemic treatment; RT, radiotherapy; SLNB, sentinel lymph node biopsy; US, ultrasonography. Colors represent compliance with minimum and target standards according to EUSOMA. Proportions and 95% confidence intervals are presented. Blue (✓✓): compliance with minimum and target standards (confidence interval contains both proportions). Green (✓): compliance with minimum but not target standards (confidence interval contains only minimum proportion). Red (✗): non-compliance with target standards (confidence interval does not contain minimum or target proportions).

**Table 7:** KPI compliance with target EUSOMA standards according to type of hospital.

	Oncology Centers	General Hospitals	*p*
	n (%)	n (%)	
Screening and diagnosis			
2.2—Proportion of women with breast cancer who preoperatively underwent: Physical examination	54 (94.7)	103 (76.9%)	0.006
3.1—Anatomopathological diagnosis: Fine-needle aspiration	5 (8.8)	43 (32.1)	0.001
3.2—Anatomopathological diagnosis: Core-needle biopsy	51 (89.5)	98 (73.1)	0.021
3.3—Anatomopathological diagnosis: Surgical biopsy	3 (5.3)	32 (23.9)	0.005
6.4—Proportion of invasive cancer cases for which the following prognostic/predictive parameters were recorded: HER2	48 (87.3)	123 (96.9)	0.019 *
7.2—Proportion of invasive cancer cases for which the following prognostic/predictive parameters were recorded: Grade	37 (92.5)	127 (100.0)	0.013 *
7.3—Proportion of invasive cancer cases for which the following prognostic/predictive parameters were recorded: ER and PgR expression	25 (58.1)	118 (92.9)	<0.001
7.4—Proportion of invasive cancer cases for which the following prognostic/predictive parameters were recorded: HER2 amplification	25 (58.1)	117 (92.9)	<0.001
7.5—Proportion of invasive cancer cases for which the following prognostic/predictive parameters were recorded: Pathological stage (pT and pN, or ypT and ypN in case of PST)	32 (74.4)	125 (98.4)	<0.001 *
7.8—Proportion of invasive cancer cases for which the following prognostic/predictive parameters were recorded: Distance to the nearest radial margin	41 (100.0)	102 (85.0)	0.007 *
8.4—Proportion of noninvasive cancer cases for which the following prognostic/predictive parameters were recorded: Distance to the nearest radial margin	20 (83.3)	16 (48.6)	0.016
Treatment			
2—Proportion of patients (invasive cancer only) who received a single (breast) surgery for the primary tumor (excluding reconstruction)	41 (71.9)	94 (90.4)	0.005
Follow-up			
2—Proportion of women with stage III breast cancer who underwent baseline staging tests (liver US, chest X-ray, or bone scan)	12 (70.6)	38 (100.0)	0.002 *

ER, estrogen receptor; HER2, human epidermal growth factor receptor 2; PgR, progesterone receptor; PST, primary systemic treatment; US, ultrasonography. * Fisher’s exact test.

**Table 8:** KPI compliance with target EUSOMA standards according to disease stage.

	Stage I	Stage II	Stage III	*p*
	n (%)	n (%)	n (%)	
Screening and diagnosis				
1—Without signs or symptoms	31 (52.5)	26 (37.7)	7 (12.5)	<0.001
10—Proportion of cancer cases preoperatively examined by MRI	20 (32.8)	41 (57.7)	38 (67.9)	<0.001
Treatment				
3—Proportion of patients with invasive cancer and clinically negative axilla who only underwent SLNB (%)	52 (96.3)	51 (92.7)	13 (68.4)	0.001
4—Proportion of patients with invasive cancer who underwent axillary clearance with excision of at least 10 nodes	6 (18.2)	17 (36.2)	36 (72.0)	<0.001
5—Proportion of patients with invasive breast cancer (M0) who received postoperative RT after primary tumor surgical resection and appropriate axillary staging/surgery in BCT settings (%)	48 (84.2)	49 (84.5)	20 (58.8)	0.006
7—Proportion of patients (excluding BRCA1 and BRCA2 patients) with invasive breast cancer no larger than 3 cm (total size, including DCIS component) who underwent BCT as a primary treatment (%)	47 (81.0)	35 (61.4)	10 (40.0)	0.001
8—Proportion of patients with invasive breast cancer who underwent axillary clearance (%)	7 (11.5)	23 (33.3)	41 (78.8)	<0.001
9—Proportion of patients with invasive breast cancer pN0 who did not undergo axillary clearance (%)	47 (87.0)	31 (86.1)	9 (60.0)	0.040

BCT, breast-conserving treatment; DCIS, ductal carcinoma in situ; MRI, magnetic resonance imaging; RT, radiotherapy; SLNB, sentinel lymph node biopsy.

## Data Availability

The dataset generated and analyzed in the current study is not publicly available due to the European Union’s general data protection regulations. They can be provided by the corresponding author upon reasonable request, after approval by the local government responsible for assessing the impact on data protection and by the study’s institutional ethics committees.
